# Sensory neuron transient receptor potential vanilloid-1 channel regulates angiogenesis through CGRP *in vivo*

**DOI:** 10.3389/fbioe.2024.1338504

**Published:** 2024-03-21

**Authors:** Zhanfeng Zhu, Yixuan Jiang, Zixia Li, Yu Du, Qinyi Chen, Qiang Guo, Yu Ban, Ping Gong

**Affiliations:** ^1^ State Key Laboratory of Oral Diseases, National Clinical Research Center for Oral Diseases, West China Hospital of Stomatology, Sichuan University, Chengdu, China; ^2^ Department of Implantology, West China Hospital of Stomatology, Sichuan University, Chengdu, China

**Keywords:** transient receptor potential vanilloid 1 (TRPV1), calcitonin gene-related peptide (CGRP), angiogenesis, vasculature, bone defect

## Abstract

Angiogenesis plays a key role in bone regeneration. The role of neurons of peripheral nerves involved in angiogenesis of bone defects needs to be explored. The transient receptor potential vanilloid 1 (TRPV1), a nociceptor of noxious stimuli, is expressed on sensory neurons. Apart from nociception, little is known about the role of sensory innervation in angiogenesis. Calcitonin gene-related peptide (CGRP), a neuropeptide secreted by sensory nerve terminals, has been associated with vascular regeneration. We characterized the reinnervation of vessels in bone repair and assessed the impact of TRPV1-CGRP signaling on early vascularization. We investigated the pro-angiogenic effect of neuronal TRPV1 in the mouse model of femur defect. Micro-CT analysis with Microfil^®^ reagent perfusion demonstrated neuronal TRPV1 activation enhanced angiogenesis by increasing vessel volume, number, and thickness. Meanwhile, TRPV1 activation upregulated the mRNA and protein expression of vascular endothelial growth factor A (VEGF-A), cell adhesion molecule-1 (CD31), and CGRP. Immunostaining revealed the co-localization of TRPV1 and CGRP in dorsal root ganglia (DRG) sensory neurons. By affecting neuronal TRPV1 channels, the release of neuronal and local CGRP was controlled. We demonstrated that TRPV1 influenced on blood vessel development by promoting CGRP release from sensory nerve terminals. Our results showed that neuronal TRPV1 played a crucial role in regulating angiogenesis during bone repair and provided important clinical implications for the development of novel therapeutic approaches for angiogenesis.

## 1 Introduction

Angiogenesis plays a key role in regenerative medicine, specifically in bone regeneration. Bone vascular system plays a significant role in bone remodeling. Due to the decrease in vascular perfusion in bone regeneration, the reduced of bone volume, bone density, osteoblast activity would be appeared, meanwhile, osteoclastic resorption would be increased (Tuckermann and Adams, 2021). Blood vessels are vital for effective bone repair as they transport oxygen, nutrients, and metabolites, and produce vascular secretion factors essential for bone growth and homeostasis (Filipowska et al., 2017).

During skeletal development and bone repair, sensory nerve fibers are strongly associated with blood vessels. It has been shown that skeletal sensory nerves play an important upstream role in the early vascularization process (Li et al., 2019; Chen et al., 2019; Xu et al., 2020). Sensory nerve fibers can transmit nociceptive signals, which become evident especially after bone fracture (Chen et al., 2019). Destructive stimuli often lead to high expression levels of the capsaicin receptor, a pain receptor, which is called transient receptor potential vanilloid 1 (TRPV1), in primary afferent neurons. TRPV1, a Ca2+-permeable, nonselective, cationic channel, is expressed in neurons of the dorsal root ganglia (DRG), trigeminal (TG) and nodose ganglia (NG), where it can be activated by heat, acidosis pH, capsaicin and other stimuli (Caterina et al., 1997; Caterina et al., 2000; Negri et al., 2020). The TRPV1 channels have been widely studied in the past, and its activity was predominantly associated with detection of noxious heat (Vandewauw et al., 2018) and the development of inflammatory hyperalgesia (Iftinca et al., 2021; Katz et al., 2023). Increasing evidence indicated TRPV1 expression in non-neuronal cells. Recent research discovered that TRPV1-mediated extracellular calcium entry by endothelial cells may increase PI3K/Akt/calmodulin-dependent protein kinase II (CaMKII) signaling to drive angiogenesis (Negri et al., 2019; Negri et al., 2020). TRPV1 is likely influential in angiogenesis, but it remains to be determined whether neuronal TRPV1 can regulate angiogenesis. Calcitonin gene-related peptide (CGRP) is one of the main sensory neuropeptides released by sensory nerve endings, which can be induced by capsaicin (TRPV1 agonist) and inhibited by capsazepine (TRPV1 antagonist) (Gao et al., 2021). CGRP is a potent vasodilator that promotes the proliferation and tube formation in endothelial cell through upregulation of VEGF expression (Mapp et al., 2012; Mi et al., 2021; Ye et al., 2021). *In vivo* studies have proven that CGRP directly stimulated angiogenesis and promoted revascularization in ischemia and wound healing by activating CGRP receptor (Toda et al., 2008; Mapp et al., 2012). It suggested that sensory nerve fibers may regulate vascular regeneration via the neuronal TRPV1-CGRP signaling axis.

In this work, we sought to investigate the role of the neuronal TRPV1 in the angiogenesis process during bone regeneration. Our hypothesis was that TRPV1-expressing afferent neurons promoted the rapid revascularization of the injury site by releasing CGRP. To test our hypothesis, we performed a series of experiments to examine the expression of TRPV1 and CGRP in the injured bone tissue and their effects on angiogenesis. Our results provided a new perspective for understanding the role of sensory nerve underlying bone repair and may have important clinical implications for the development of novel therapeutic approaches for angiogenesis.

## 2 Methods

### 2.1 Experimental animals

All experiments were approved by the Ethics Committee of West China Hospital of Stomatology (Chengdu, China). All experiments were performed in accordance with relevant guidelines and regulations and executed in compliance with the ARRIVE guidelines (https://arriveguidelines.org). Briefly, 8–10-week-old, male, C57BL/6 mice (20–25 g) were provided by Dossy Experimental Animals Company (Chengdu, China). The animals were exposed to a 12-h light-and-dark cycle. Mice were allowed for a standard diet with free access to food and water. The mice were randomly allocated into the following four groups: control group that consisted of 31 mice (*n* = 31) receiving phosphate-buffered saline injection; capsaicin group that consisted of 31 mice (*n* = 31) receiving capsaicin injection; capsazepine group that consisted of 31 mice (*n* = 31) receiving capsazepine injection; capsazepine + CGRP group that consisted of 28 mice (*n* = 28) receiving capsazepine and CGRP overexpression lentivirus injection.

### 2.2 Surgical procedure

A mouse model of bone defect was established in accordance with a previous study (Monfoulet et al., 2010). General anesthesia was administered using a mixture of 80 mg/kg ketamine-HCl and 10 mg/kg xylazine HCL intraperitoneally. The animal was fixed with the prone position, and the fur on distal surface of right and left legs was shaved and disinfected with povidone-iodine. Reaching the distal surface of the femur, the soft tissue was separated, the periosteum was released, and the bone was exposed. A standard bone defect measuring 1-mm in diameter was created using round drills in both the right and left femur of each mouse. The mice were killed post-surgery either on day 7 or day 14. The femurs were isolated for subsequent analysis.

### 2.3 Drug injection

The drug was administered intrathecally to mice by direct lumbar puncture as described previously (Pan et al., 2018). Control group received topical applications of PBS. For mice in capsaicin group, lumbar puncture was performed using a 5-μL Hamilton microsyringe (Hamilton, United States) fitted with a 30-G needle to deliver 2 μL capsaicin (2 mg/mL, Abmole, United States) into the subdural space of the spinal L4–L6 levels in non-anaesthetized mice before surgery (Jiang et al., 2023). For mice in capsazepine group, 0.5 μL capsazepine (2 mg/mL, Abmole, United States) was injected by lumbar puncture per day. Successful puncture was indicated by a reflexive lateral flick of the tail. For mice in capsazepine + CGRP group, CGRP overexpression lentivirus 10 μL (2.88 × 10^8^ TU/mL, GeneCopoeia Co., China) was injected around the femoral bone defect locally.

### 2.4 Retrograde tracing of DRG neurons

Using a 30-gauge needle, we injected 0.5-µL Fluoro-gold (FG, Fluorochrome, CO, United States) into the bone defect area to retrogradely label DRG neurons by a Hamilton microsyringe (Hamilton, United States). After 48 h, L4-L6 DRGs were harvested for immunofluorescence (IF) staining of DRG sections.

### 2.5 Microangiography

After general anesthesia, the skin was separated from the neck down to the glabella. The chest was opened and expose the heart. The right atrium was clipped. 100 U/mL heparin solution, 4% paraformaldehyde and Microfil^®^ reagent (MV-122, Flow Tech Inc, United States) were perfused successively via the left ventricle and incubation overnight at 4°C. The femurs were stripped, fixed in 4% paraformaldehyde for 48 h and decalcified in 10% EDTA for 4 weeks, and then scanned for micro-CT.

### 2.6 Micro-CT analysis

For Micro-CT analysis, four animals were used at each timepoint in each group. The perfused femurs were imaged on a micro-CT (SCANCO 50, Switzerland) using 10-μm resolution with a voltage of 90 kV and a current of 88 μA. The region of interest (ROI) was defined as the defect of the femurs to evaluate the vascular microstructure. Vascular volume (VV), vascular volume fraction (VVF), vascular number (V*N), vascular thickness (V*Th), and vascular separation (V*Sp) were calculated to evaluate vascularization of the defect.

### 2.7 Immunohistochemistry evaluation

For Immunohistochemistry evaluation, five animals were used at each timepoint in each group. Femurs were fixed in 4% PFA for 48 h and decalcified in 10% EDTA (pH 7.0) for 4 weeks. The samples were dehydrated in ethanol concentrations (70%–95%), washed in xylene and embedded in paraffin blocks to obtain 5 μm thick cross-sections. After deparaffinization, immunohistochemistry staining was done. To prevent non-specific binding, 3% bovine serum albumin (BSA) was incubated with all sections for 30 min. Sections were incubated with primary antibodies, anti-CGRP (1:100, Santa Cruz, United States), anti-CD31 (1:100, Santa Cruz, United States), and anti-alpha smooth muscle actin (α-SMA) (1:100, Santa Cruz, United States) overnight at 4°C. Sections were rinsed three times with PBS before being incubated for 1 h at room temperature with HRP labeled secondary antibody (1:500, Abcam, United States). Finally staining was shown with diaminobenzidine (DAB) substrate, followed by counterstaining with a hematoxylin. The mean optical densities (Integral optical density/area of interest) were calculated by ImageJ 1.46 software (NIH, United States) for further semi-quantitative analysis of CD31, a-SMA and CGRP expression.

For immunofluorescence, L4-L6 DRGs were extracted after injecting retrograde tracer for 72 h and fixed in 4% paraformaldehyde for 24 h and embedded in paraffin. Sections were incubated for 20 min at room temperature in a blocking solution containing 3% BSA. Sections were incubated with primary antibody anti-TRPV1 (Santa Cruz, United States) overnight at 4°C and incubated with Cy3 labeled secondary antibody (1:100, Servicebio, China) for 50 min at room temperature, then incubate in TSA (1:500, Servicebio, China) for 10mins at room temperature in the dark. After antigen retrieval with microwave oven for 10 min, second primary antibody, anti-CGRP (Santa Cruz, United States) was applied overnight at 4°C and incubated with FITC labeled secondary antibody (1:100, Servicebio, China) for 50 min at room temperature.

### 2.8 qRT-PCR (quantitative real-time PCR)

Bone tissue from the distal and proximal 1 mm of the femoral defect and L4-L6 DRGs from five animals in each group were collected at days 7 and 14. Total RNA was isolated from tissues and DRGs using the FastPure Cell/Tissue Total RNA Isolation Kit V2 (Vazyme, China). RNA was reversely transcribed into cDNA using HiScript III RT SuperMix for qPCR (+gDNA wiper) (Vazyme, China) and amplified using Taq Pro Universal SYBR qPCR Master Mix (Vazyme, China). Real-time PCR was carried out through a LightCycler 480 system (Roche Diagnostics, Germany). Glyceraldehyde-3-phosphate dehydrogenase (GAPDH) was used as the internal control for normalization. The primer sequences were listed in Table 1.

**TABLE 1:** Sequences of the primers used for RT-qPCR.

Gene	Forward (5′-3′)	Reverse (5′-3′)
Trpv1	TTC​ACC​ACG​GCT​GCT​TAC​TA	CCA​CAG​ACA​CCA​GCA​TGA​AC
Cgrp	TCT​CCC​TTT​GAC​AGG​AGC​TAA​A	ATG​TGT​CCC​CAG​AAG​AGC​AAG
Cd31	AAA​TCA​AGC​CCC​CTG​GGA​TG	GAG​GTG​GCT​ACA​ATC​GCC​TT
Vegfa	GAC​TAT​TCA​GCG​GAC​TCA​CCA	TGA​GGG​AGT​GAA​GAA​CCA​ACC
Gapdh	CCT​CGT​CCC​GTA​GAC​AAA​ATG	TGA​GGT​CAA​TGA​AGG​GGT​CGT

### 2.9 Western blot

Proteins of specimens from three mice in each group were extracted by RIPA buffer (Sigma-Aldrich, United States) followed by high-speed centrifugation (12,000 g/min at 4°C for 10 min) at day 7. The protein concentration was determined using a BCA protein quantitative kit (Beyotime, China). 20 μg of total protein was separated by SDS-PAGE, electrophoresed, and transferred onto a PVDF membrane (Sigma-Aldrich, United States). The membrane was blocked with 5% BSA/TBST for 1 h, then incubated with the following primary antibodies: anti-CGRP (1:1,000, Santa Cruz, United States), anti-VEGFA (1:1,000, Abcam, United States) and anti-GAPDH (1:5,000, Signalway Antibody, United States) at 4°C overnight. The membrane was washed with TBST thrice and then incubated with HRP labeled secondary antibody (1:5,000, Abclonal, China) for 2-3 h at room temperature. The membrance was treated with the ECL blotting reagents (Zenbio, China). The protein bands were visualized with QuantityOne software (Bio-Rad, United States). Before incubating other primary antibody, the blots were rinsed in distilled water for 5 min, then soaked in the antibody stripping buffer (Servicebio, China) for 60 min to remove ECL substrates.

### 2.10 Statistical analysis

All data were expressed as mean ± SD. Statistical differences were determined using one-way ANOVA with Tukey’s multiple comparison test, *p* < 0.05 was considered statistically significant. Graphs were produced by GraphPad Prism Software (GraphPad Prism 9.0, United States).

## 3 Results

### 3.1 Expression and inhibition of TRPV1 and CGRP in DRG sensory neurons

**FIGURE 1:**
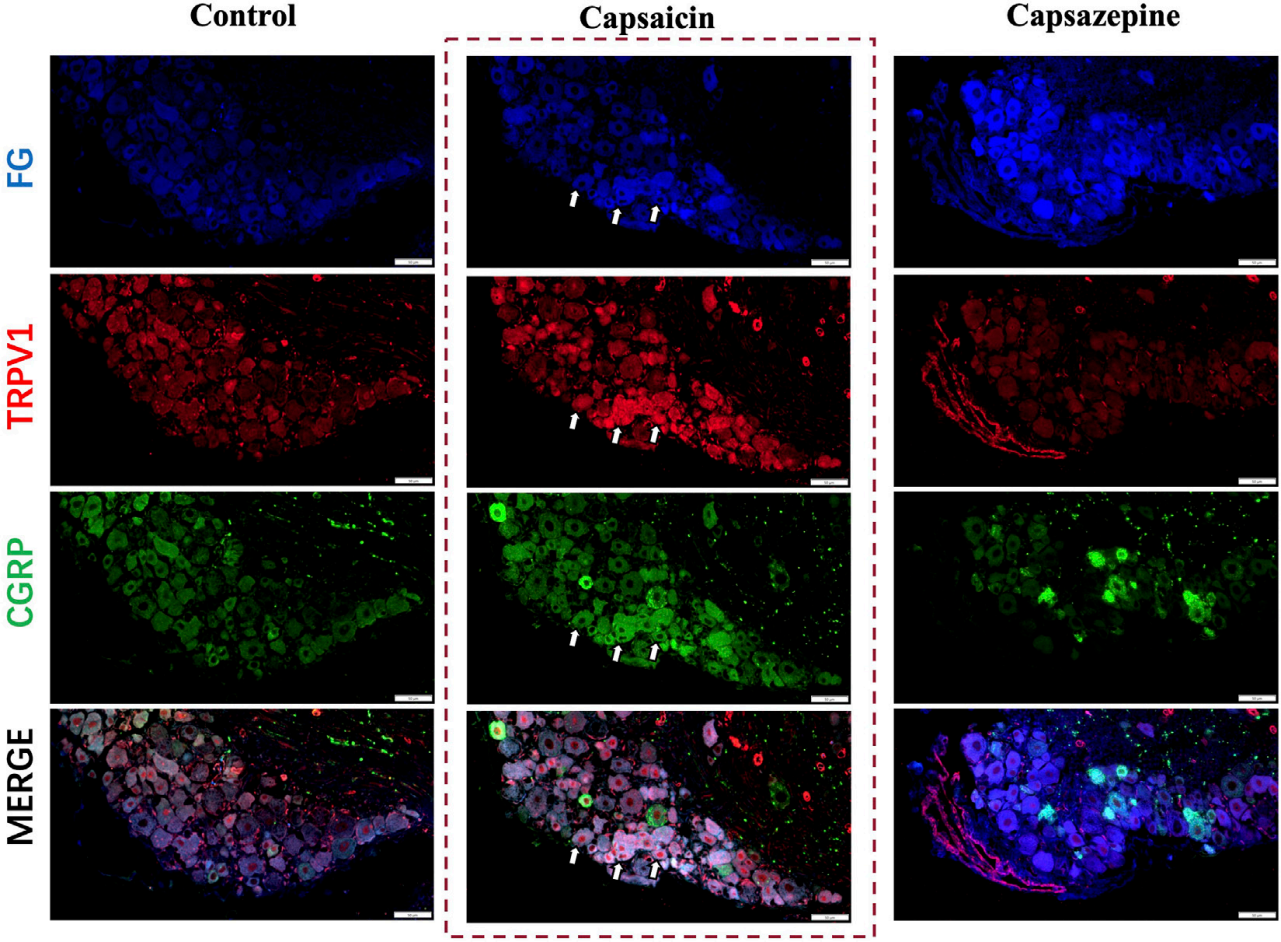
Retrograde tracing of DRG neurons on day 7 after surgery. Retrograde labelled Fluoro-gold (FG) and immunofluorescence staining of TRPV1 (Red) and CGRP (Green) in DRG sensory neurons (Blue). The arrows indicate FG-positive neurons co-expressing TRPV1 and CGRP. Scale bar = 50 μm.

Capsaicin serves as a potent agonist of the TRPV1 channel. This experiment found the same results in that capsaicin activated the TRPV1+ sensory neurons in the DRG (Caterina et al., 2000; Li et al., 2019; Lee et al., 2020). To further investigate the potential co-expression of TRPV1 and CGRP in DRG nerve fibers, Fluoro-gold (FG) was injected at the defect to retrograde label the upstream neurons in the DRG and we conducted immunofluorescence staining (Figure 1). Our findings indicated that TRPV1 (Red) immunolabeling and CGRP (Green) expression significantly overlapped in the FG-positive neurons (Blue), indicating that DRG nerve fibers express both CGRP and TRPV1. After activating through capsaicin, both TRPV1 and CGRP expression were elevated in the neuron. Furthermore, Capsazepine, a TRPV1 antagonist, the expression of TRPV1 and CGRP were weakened simultaneously in comparison to the control group. Taken together, these findings suggest that TRPV1 and CGRP are co-expressed in DRG nerve fibers, and capsaicin enhanced the expression of both TRPV1 and CGRP.

### 3.2 Effects of neuronal TRPV1 activation and inhibition on vascular regeneration

**FIGURE 2:**
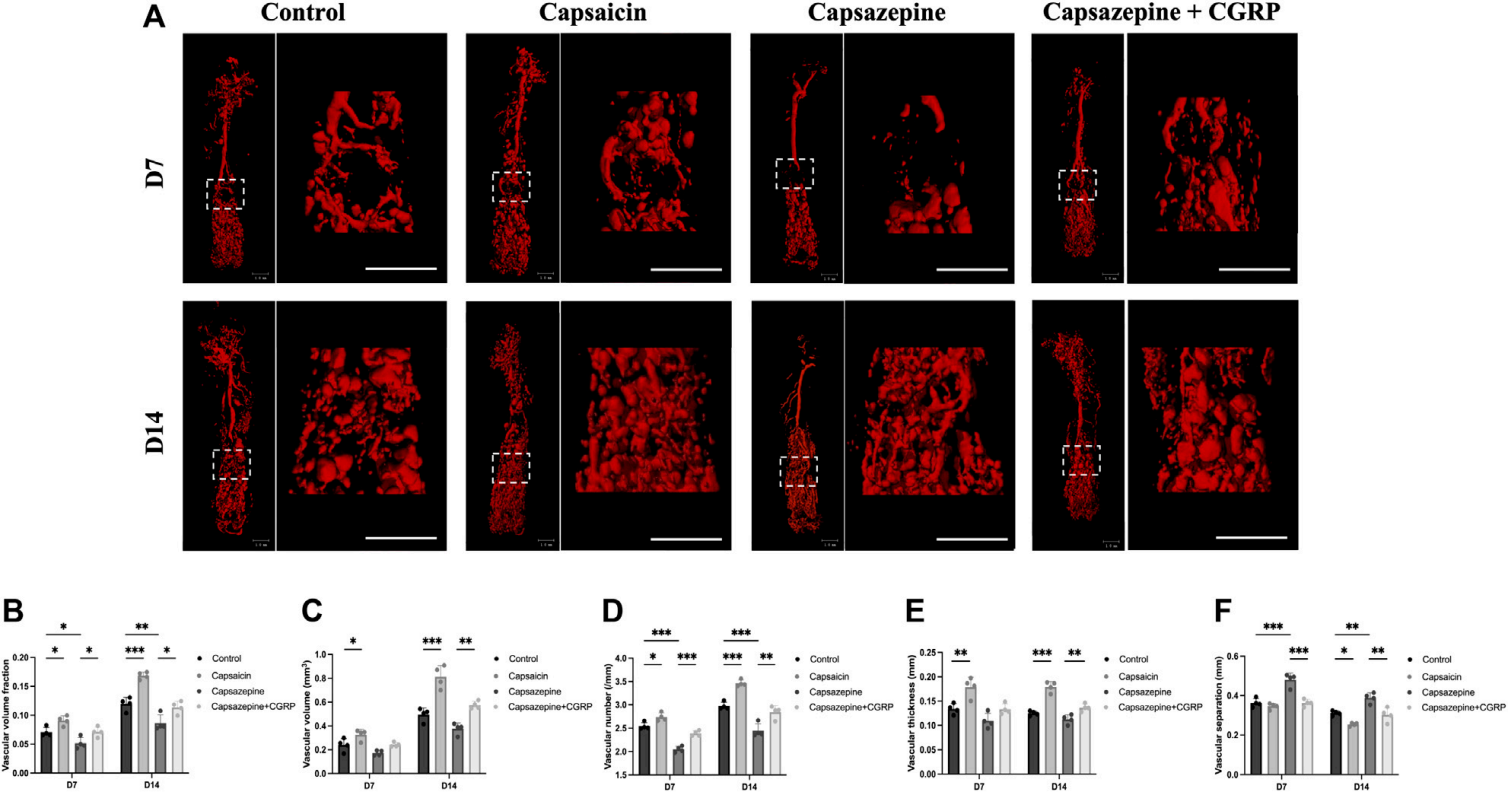
The activation and inhibition of TRPV1 effects on vascular regeneration in bone defects. **(A)** Micro-CT 3D reconstructed pictures of the femur vasculature on days 7 and 14 from four groups. Scale bar = 1 mm. **(B–F)** The quantitative evaluation of the vascular morphology with respect to vascular volume fraction (VVF), vascular volume (VV), vascular number (V.N), vascular thickness (V.Th) and vascular separation (V.Sp). (*n* = 4). The data are expressed as the mean ± SD. *p < 0.05, **p < 0.01 and ***p < 0.001 compared with the control.

To observe the vascular formation in a femur defect, mice were perfused with Microfil^®^ reagent. Multi-perspective 3D-reconstructed images revealed that the capsazepine group had less and thinner vessels, with decreased interconnectivity and increased separation compared to the control group; however, the capsaicin group showed the opposite result (Figure 2A). Quantification of the microstructural parameters revealed that the VVF, V.N, V.Th, and VV in the defect were higher in the capsaicin group than the control group, while the V.Sp was significantly lower than the control group V.Sp, lower V.Sp represented that vessels were closer to each other, and the area was more densely vascularized. The capsazepine group showed lower levels of the VVF, V.N, V.Th, and VV and a higher level of V.Sp than the control group. We restored CGRP levels in localized bone defect by injecting CGRP lentiviral vector after capsazepine injection. The local CGRP rescue treatment enhanced vascularization in femur defect. The level of VVF, V.N, and V.Th was improved and V.Sp was lower than the capsazepine group (Figures 2B–F). These results suggest that activation of TRPV1 by capsaicin improves vascular formation, while capsazepine reduces it. These findings have significant implications for understanding the role of TRPV1 in vascular formation.

### 3.3 Activation of neuronal TRPV1 promotes the expression of proteins related to local vascular regeneration

**FIGURE 3:**
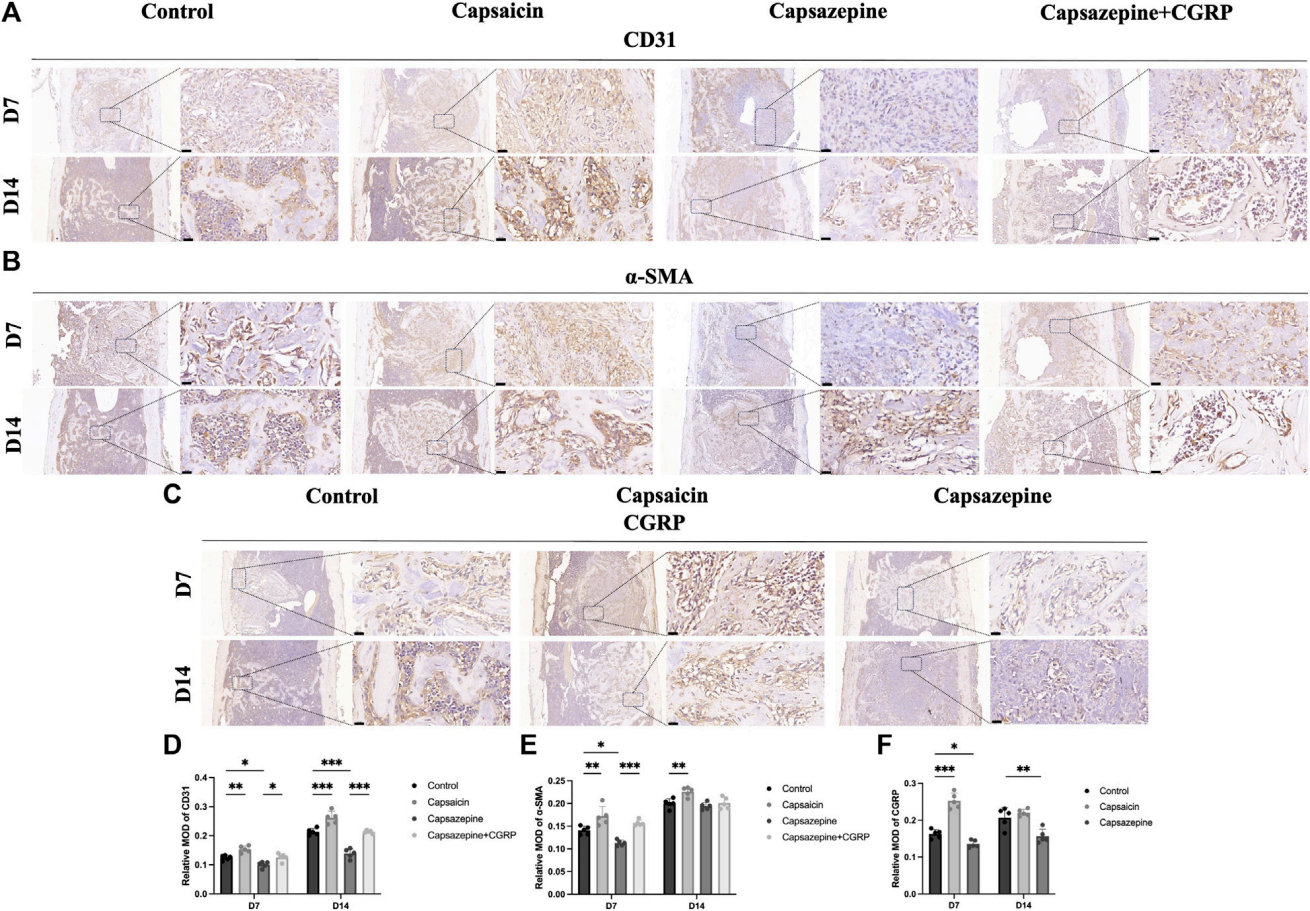
Bone-defect angiogenesis was related to upregulated TRPV1 and CGRP. **(A–C)** Immunohistochemical staining of CD31, a-SMA and CGRP in femur bone defect. Scale bar = 20 μm. **(D–F)** Semiquantitative analysis of CD31, a-SMA and CGRP expression in the defect area. MOD (mean optical densities) = Integral optical density/Area. (*n* = 5). *p < 0.05, **p < 0.01 and ***p < 0.001 compared with the control.

The immunohistochemical assay was conducted to observe the expression of CD31, α-SMA, and CGRP in newly formed tissue (Figures 3A–C). The intensities of CD31 and α-SMA immunostaining were substantially greater in the capsaicin group than in the control group, but significantly lower in the capsazepine group. Local rescue by CGRP promoted CD31 and α-SMA expressions. Moreover, the CD31 and α-SMA intensities continuously increased from 7 to 14 days in each group, while the trend of differences between groups remained consistent (Figures 3D, E). In addition, CGRP was strongly expressed in the capsaicin group, but showed lower expression in the capsazepine group than the control group. Interestingly, CGRP expression was lower in the capsaicin group at day 14 than at day 7 but was still the highest among all three groups (Figure 3F). These findings suggest that the TRPV1 agonist regulates angiogenesis of the bone defect area through its downstream gene *CGRP in vivo*.

### 3.4 Neuronal TRPV1 upregulated the expression of local angiogenic genes

**FIGURE 4:**
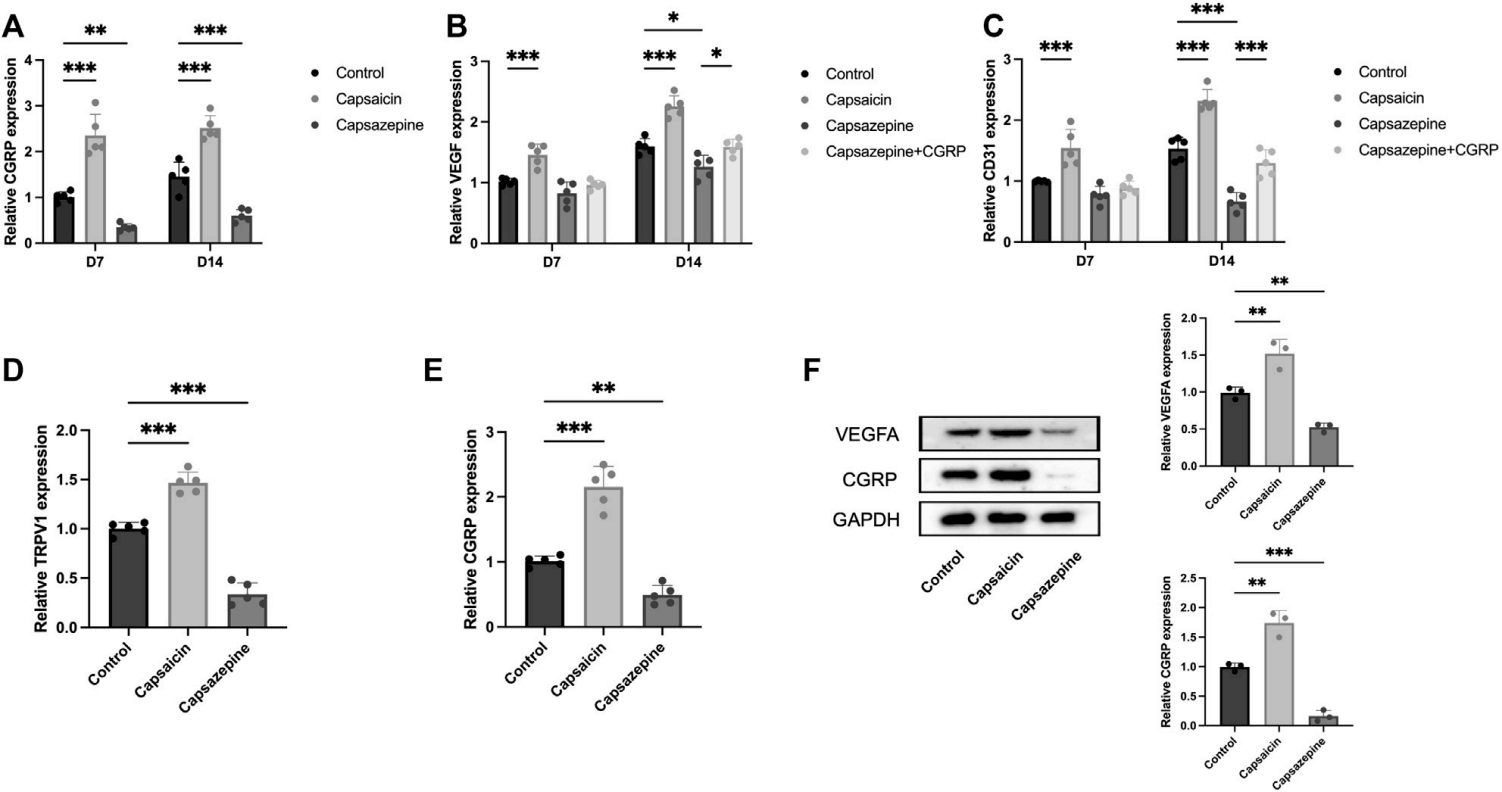
The activation and inhibition of TRPV1 gene in bone defect and DRG neuron. **(A)** PCR analysis of CGRP mRNA expression in defect region tissues of three groups on days 7 and 14. (*n* = 5) **(B)** PCR analysis of VEGF mRNA expression in defect region tissues of four groups on days 7 and 14. **(C)** PCR study of CD31 mRNA expression in defect region tissues of four groups on days 7 and 14. **(D)** PCR analysis of TRPV1 mRNA expression in DRG sensory neurons of three groups. **(E)** PCR analysis of CGRP mRNA expression in DRG sensory neurons of three groups. **(F)** Western blot analysis of CGRP and VEGFA expression in the defect of three groups (*n* = 3). Original full-length gels are presented in Supplementary Material. All data are shown as the as the mean ± SD. *p < 0.05, **p < 0.01 and ***p < 0.001 compared with the control.

To evaluate the expression of angiogenesis-related genes, we extracted mRNA and protein from bone tissue surrounding the defect. Specifically, we analyzed the expression of CGRP, VEGF, and CD31 in these samples. PCR results showed a significant upregulation in the capsaicin group, whereas the capsazepine group exhibited a downregulation of CGRP, VEGF, and CD31 mRNA expression. After local injection of CGRP lentivirus, the VEGF and CD31 mRNA expression level was elevated in the capsazepine group (Figures 4A–C). The expression of CGRP, VEGF, and CD31 continuously increased from 7 to 14 days in each group, while the trend of differences between groups remained consistent. The result of western blot demonstrated that CGRP and VEGF protein expressions were simultaneously increased in the capsaicin group and both decreased in the capsazpine group (Figure 4F). Meanwhile, sensory neurons in DRG were also extracted. The results demonstrated that TRPV1 inhibition exhibited a co-repressive effect on mRNA expression of TRPV1 and CGRP, while the activation of TRPV1 showed the positive effect (Figures 4D, E). Taken together, this suggested that neuronal TRPV1 could regulate the expression of angiogenic genes in defect and possibly relate to CGRP expression.

## 4 Discussion

Tissue vascularization plays a critical role in the regeneration of bone defects (Kusumbe et al., 2014). However, the function of TRPV1 activation in angiogenesis has not been well studied in the literature. In our previous research, we demonstrated that CGRP not only promotes peri-implant vascular regeneration, but bone regeneration in the defect (Guo et al., 2020). In the present study, we investigated the effect of TRPV1 activation in DRG neurons, which evoked the release of the neuropeptide CGRP, leading to improve vessel formation in a mouse model of femur defect. Our research findings suggested that the blockage of TRPV1 with capsazepine in DRG sensory neurons led to a decrease of local vascular regeneration. The results of qPCR and western blotting of representative angiogenesis and endothelial tissue markers including VEGF, CD31, and α-SMA confirmed the facilitation of angiogenesis by TRPV1 activation. However, the capsazepine group showed the opposite effect. We were surprised to find that changes in CGRP expression were the same as changes in angiogenic markers in both DRG and the defect, suggesting that regulating neuronal TRPV1 could improve the expression of both CGRP and angiogenic factors. With our previous study about CGRP promoting vascular regeneration, the results suggested that stimulating neuronal TRPV1 might be able to promote angiogenesis through releasing CGRP.

Immunostaining for CD31 revealed a stronger positive reaction in the capsaicin group than in the control group, indicating that TRPV1 activation provided endothelial cells with information related to angiogenesis and promoted mature blood vessels formation. High expression of CD31 represented an increasing number of the formation of H-Type-like vessels, a specialized capillary subtype associated with osteogenesis by providing molecular signals acting on osteoprogenitor cells (Kusumbe et al., 2014; Peng et al., 2020; Zhang et al., 2020). The defect undergone a hematoma and an acute inflammatory phase followed by the formation of granulation tissue, which was rich in proliferating mesenchymal cells and capillaries (Loi et al., 2016). Blood vessels provided an excellent source of osteoprogenitor cells, which were distributed around H-type vessels. With expressing osteogenic factors Osterix and Runx2, osteogenic progenitor cells could promote bone formation (Claes et al., 2012). Granulation tissue formed the basis for the later aggregation of chondrocytes and the calcification of chondrocytes. Therefore, the activation of TRPV1 might promote angiogenesis and partially contribute to stimulating proliferation and differentiation of osteoprogenitor cells in bone regeneration. This study provided insights into the role of TRPV1 in angiogenesis during bone regeneration.

Recent studies have reported that endothelial TRPV1 stimulates angiogenesis through TRPV1-mediated extracellular calcium entry, promoting PI3K/Akt/CaMKII signaling (Negri et al., 2019). (Ching et al., 2011) revealed that activating endothelial TRPV1 promoted the formation of the TRPV1-eNOS complex, thus promoting NO releasing and enhanced VEGF secretion, resulting in angiogenesis. Although endothelial TRPV1 has been shown to promote angiogenesis (Lee et al., 2021), TRPV1 was primarily enriched in small- and medium-diameter sensory neurons within the DRG (Caterina et al., 1997). The mechanism by which TRPV1 of DRG sensory neurons enhanced angiogenesis has not been fully elucidated. (Xiang et al., 2017). To partially address this, we focused on the role of sensory neuron TRPV1 in the angiogenesis of bone regeneration. To avoid the effects of TRPV1 channels at other locations, drugs were delivered intrathecally by direct lumbar puncture to inhibit or activate TRPV1 channels directly in the neurons. The results demonstrated that the activation of sensory neuron TRPV1 enhanced angiogenesis. After conducting our previous research on the role of CGRP in vascular regeneration (Claes et al., 2012; Loi et al., 2016; Li et al., 2019), we evaluated the expression levels of CGRP at the bone defect and found that the activation of TRPV1 resulted in a significant increase in CGRP expression. Conversely, the use of a TRPV1 antagonist in DRG led to a significant decrease in CGRP expression. Based on these data, we speculated that activating TRPV1 channels in sensory neurons promoted the production of angiogenic factors, such as VEGF, by releasing the neurotransmitter CGRP.

(Zhang et al., 2016) reported that magnesium promoted angiogenesis in bone formation via promoting CGRP synthesis in DRG. Meanwhile, (Mi et al., 2022) discovered that by electrically stimulating DRG thereby triggering a rapid release of CGRP stored in nerve terminal vesicles, H-vessel formation was promoted at the femoral fracture site. Stimulation of TRPV1 at nerve ending induced exocytosis following neuronal depolarization, causing synaptic vesicles to secrete outwardly (Fields et al., 1997). Our findings showed that CGRP expression similarly raised after activation of TRPV1 using capsaicin, suggesting that TRPV1 promoted releasing CGRP via exocytosis following neuronal depolarization, rather than being released directly. This mechanism appeared to be critical for the angiogenesis-osteogenesis coupling during bone regeneration (Mishima et al., 2011). These findings were similar to prior research that have revealed that inhibiting TRPV1 affected CGRP expression and co-expression in TG, which was compatible with the findings of our investigation (Takahashi et al., 2016; Wang et al., 2018; Guo et al., 2020). Therefore, our results suggested that angiogenesis induced by TRPV1 was depended on the regulation of CGRP and its relevant pathways. Our findings showed that TRPV1 regulated CGRP expression and thereby played an important role in vascular regeneration. Stimulation of neuronal TRPV1 could elevate endogenous CGRP synthesis and release to induce angiogenesis in bone defect.

The infusion of high dose of capsaicin lead to a profound and sustained desensitization of the affected nerve fibers due to intracellular calcium overload (Arora et al., 2022). Fiber desensitization was a protective mechanism to inhibit excess calcium influx, leading to degeneration, retraction or even excitotoxic cell death (Wang et al., 2018). Although activation of TRPV1 in peripheral nerve using high concentration of capsaicin could lead to the ablation of TRPV1, the dose we used was insufficient to desensitize the nerve fiber, much less the concentration that cause nerve damage, but could activation the neuronal TRPV1.

In this study, we aimed to investigate the effects of manipulating the TRPV1 channel of sensory neurons on angiogenesis. Our results showed that intrathecal injection of capsaicin at DRG enhanced angiogenesis in mouse femoral defects. We showed that TRPV1 affected vascular formation by promoting the release of CGRP from sensory nerve terminals. However, one drawback of our work was the absence of examination into the mechanisms of TRPV1 in angiogenesis *in vitro*. Further investigations were necessary to determine the impact of neuronal TRPV1 on the proliferation and differentiation of endothelial cells via CGRP. Additionally, the effect of TRPV1 on osteogenesis in bone defects required further exploration. In future studies, we plan to focus on these aspects. Nevertheless, the findings of our study provided a basis for the therapeutic approach toward bone defects, by taking advantage of the activation of TRPV1 to effectively enhance angiogenesis and regenerate bone tissue.

## 5 Conclusion

Our study sheds light on the crucial role of TRPV1-positive sensory neurons in regulating angiogenesis during bone repair. We have shown that TRPV1 activation leads to increased expression of CGRP, which contributes to promoting angiogenesis. Overall, our findings indicated that the TRPV1-CGRP signaling axis could be a suitable target for the development of novel therapies aimed at promoting angiogenesis, which provided an idea for the clinical treatment of bone repair through sensory nerve fibers modulation of vascular regeneration.

## Data Availability

The datasets presented in this study can be found in online repositories. The names of the repository/repositories and accession number(s) can be found in the article/Supplementary Material.
