# Supplementary material for: Sensory neuron transient receptor potential vanilloid-1 channel regulates angiogenesis through CGRP in vivo
Source: Front Bioeng Biotechnol. 2024 Mar 21;12:1338504. doi: 10.3389/fbioe.2024.1338504 (PMC10991839; doi:10.3389/fbioe.2024.1338504)
Supplement: Supplementary file 1 [file DataSheet1.PDF]

Supplementary data

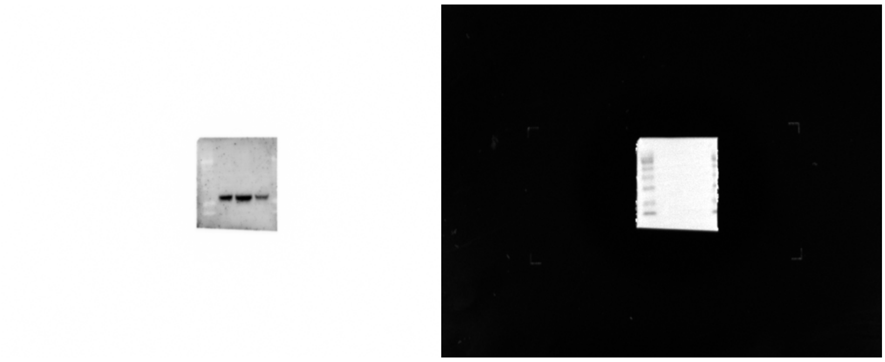

Figure S1.2. Full-length gel of VEGFA expression (multiple exposures) and the blot that washed by stripping buffer. Molecular size: 25-35 kDa

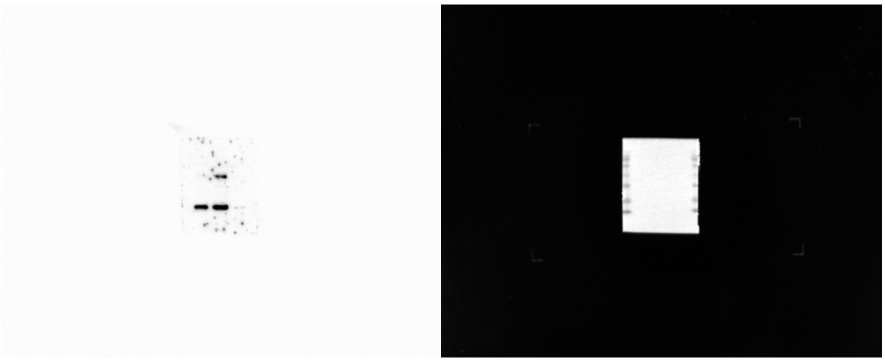

Figure S3.4. Full-length gel of CGRP expression (multiple exposures) and the blot that washed by stripping buffer. Molecular size: 15 kDa

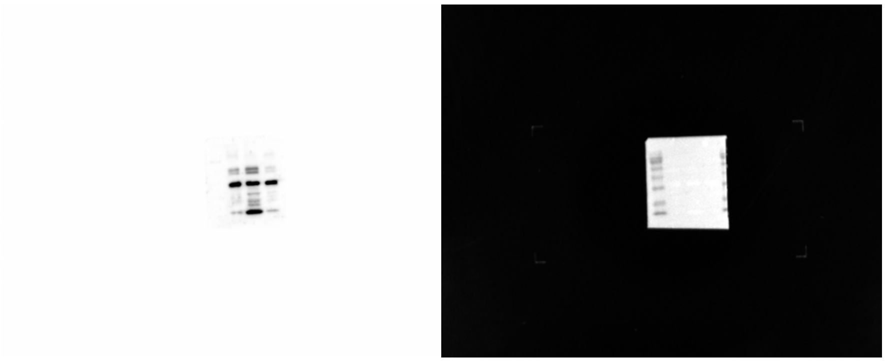

Figure S5.6. Full-length gel of GAPDH expression (multiple exposures) and the blot that washed by stripping buffer. Molecular size: 35 kDa
